# House Dust Mite Prevalence in the House of Patients with Atopic Dermatitis in Mashhad, Iran

**Published:** 2017-05-27

**Authors:** Toktam Ziyaei, Fariba Berenji, Farahzad Jabbari-Azad, Abdolmajid Fata, Lida Jarahi, Mohammad Fereidouni

**Affiliations:** 1 Department of Parasitology, School of Medicine, Mashhad University of Medical Sciences, Mashhad, Iran; 2 Department of Immunology and Allergy, Head of Allergy Research Center, Ghaem Hospital, Mashhad University of Medical Sciences, Mashhad, Iran; 3 Department of Community Medicine, School of Medicine, Mashhad University of Medical Sciences, Mashhad, Iran; 4 Department of Immunology, Asthma, Allergy and Immunology Research Center, Birjand University of Medical Sciences, Birjand, Iran

**Keywords:** House dust mite, Atopic dermatitis, *Dermatophagoides*, Iran

## Abstract

**Background:**

Being exposed to house dust mites intensifies atopic dermatitis. This study has investigated the contamination rate with *Dermatophagoides* mites in patient’s residential home with atopic dermatitis.

**Methods:**

In this cross-sectional study, 40 patients took part with atopic dermatitis (positive or negative for mites by prick Dermal Test). Samples were collected from 3 locations (living room, bedroom and bed) by vacuum cleaner. Dust samples (transferred to freezer −20 °C) were examined by direct method and flotation. The data were analyzed using statistical SPSS vr.20 software.

**Results:**

Twenty patients of positive prick test included 8 (40%) male and 12 (60%) female. The results of direct observation of mites: 7 cases (35%) in bedding sheets, 6 cases (30%) bedrooms’ carpet, 3 cases (15%) living room’s carpet. Twenty patients of negative prick test included 8 (40%) male and 12 (60%) female. Only mites were found (5%) in living room’s carpets of negative prick test patients. *Dermatophagoides pteronyssinus* was more frequent than *Dermatophagoides farina*e. (98% vs 83%).

**Conclusion:**

Fifty-five percent of residential homes of prick test positive patients and only 5% of residential homes of prick test negative patients were positive for mite. Sunshine provided home had fewer mites than home where sunshine is not provided. Prick test positive patients used handmade carpets more than machine made ones. In positive prick test patients, mites were found in bed sheet and bedroom’s carpet more than negative prick test patient’s sheets and carpets.

## Introduction

House dust mites are eight-legged creatures with about 1.3mm in length and are not visible by naked eyes (Platts-Mills and Chapman 1987). They belong to phylum Arthropoda, class Arachnida, subclass Acarina order Acariformes and Pyroglyphidae family. This family has about 16 and 46 species. Three species called *Dermatophagoides pteronyssinus*, *De. farina* and *Euroglyphus maynei* are more important and are the source of allergy resulted from mites (Arlian and Platts-Mills 2001). House dust mites exist in the dust all around the world but they are mostly found in bed sheets, carpets, furniture and cloth toys (Platts-Mills and Chapman 1987, Babe et al. 1995, Beltrani 1997, Arlian and Platts-Mills 2001, Sidenius et al. 2002, Mihrshahi et al. 2002, Yong and Jeong 2009).

In 1g of the dust collected from a carpet, more than 10000 mites have been counted. On average, 500 mites have been collected out of 0.2g of dust (Nadchatram 2005). These arthropods feed on outer layer of human and animal skin and other organic materials (Platts-Mills and Chapman 1987, Beltrani 1997, Nadchatram 2005, Yong and Jeong 2009). They reproduce sexually and their development cycle to maturity includes 5 steps: eggs, larva, protonymph, tritonymph, and adult (Arlian and Platts-Mills 2001). It takes about 30d for dust mites to complete their life cycle (Nadchatram 2005, Yong and Jeong 2009).

The rate of mite growth depends on humidity and temperature. The best environmental condition for reproduction is the temperature of 23 °C and the relative humidity of 70 % (Platts-Mills and Chapman 1987, Babe et al. 1995, Arlian and Platts-Mills 2001). Mite excretion and skin frill are the allergen factors in human (Blythe 1976, Platts-Mills and Chapman 1987, Arlian and Platts-Mills 2001, Cui 2014). There are a lot of evidences on the importance of mite allergens to the etiology of allergic asthma, non-seasonal allergic rhinitis and atopic dermatitis (Platts-Mills and Chapman 1987, Arlian and Platts-Mills 2001, Engelhart et al. 2002, Nadchatram 2005, Jacquet 2011).

Dermatitis or eczema is a kind of inflammatory reaction of skin to different factors. Its clinical symptoms include itch, erythema, scaling, often in the flexor surfaces of the body (Berke et al. 2012). It happens mostly to children (Fuiano and Incorvaia 2012). This disease usually begins in the early life and is often seen in the people with the personal or family background of asthma or swelling of the mucous membranes (Fuiano and Incorvaia 2012). Forty five percent of children experience the early onset in the first 6 months of their life, 60% during their first year and 85% before the age of 5 (Fuiano and Incorvaia 2012). This disease can assist with asthma and allergic rhinitis (Berke et al. 2012). This disease can cause sleep, educational, and social disorders in patients (Hanifin and Rogge 1977, Queille-Roussel et al. 1985, Laughter et al. 2000). Moreover, the physical and psychological pressure can effect on not only the patients but also their families and related people (Lapidus et al. 1993, Herd et al. 1996).

According to the importance of home dust in relation to atopic dermatitis in Iran and especially in Mashhad has been studied lesser, the present study has been conducted in order to determine the abundance of *Dermatophagoides* mites in the houses of patients suffering from atopic dermatitis.

## Materials and Methods

### Statistical population

The present study is a cross-sectional study. Forty patients with atopic dermatitis, based on defined criteria for the disease, have been presented to the Allergy Clinic of Ghaem Hospital of Mashhad, Iran. Their disease had been diagnosed by dermatologists based on clinical criteria. They took part in the study after giving their written consent. The study was approved by the local Ethics Committee.

Before doing Prick test, the necessity and method of doing the test was explained to the patients. For each patient, some information like age, sex, home description, job, allergy background of the patient or his/her family was recorded in a questionnaire. Considering no drug interaction with dermal tests, a dermal test was done. Some international standard extracts including mite allergens (Derf1, Derp1) assisting with positive control (Histamine) and negative control with normal saline, were put on the patients’ forearms, on the points at a distance of 2cm from each other. Then the skin was scratched with a lancet. If the skin bumps were bigger than 3mm, the patient was referred to as sensitive to mites and if the bumps were smaller than 3mm, he/she was referred to non-sensitive to mites. The patients, who did positive or negative dermal test for mite allergens, participated in the study.

### Dust samples

Before sampling, the patients were asked to avoid vacuuming or sweeping mats in their houses or changing their bed sheets for 3 to 5d. Then referring to the patients’ houses separately, samples were collected from 3 locations (living room, bedroom and bed) by vacuum cleaner on high power for 2 minutes and in an area of 1m^2^. Then the samples were transmitted to special bags and the bags containing dust were closed and the specifications relating to locations, dates, humidity amount and temperature were written on the bags and the samples was frozen at −20 °C.

### Flotation

Dust samples were weighed using a 0.001 g digital scale and it was separated into 100 mg aliquots. Mites were isolated using a flotation method (Fernandez-Caldas et al. 1993). Briefly, dust samples were suspended in 5ml of saturated saline and sample were examined under the light microscope using 10–40 × magnification.

### Statistical analysis

Statistical analysis was performed using SPSS software for Windows, ver.20 (SPSS, IBM, USA). Chi-square test, Fisher Exact Test was used to evaluate the variables correlations. Furthermore, the significant statistical level in these tests was considered with P< 0.05.

## Results

Forty patients were evaluated who had atopic dermatitis, 24 (60%) were female and 16 (40%) were male. They were between 5 month and 45yr old (mean age 16.91yr).

In terms of occupation, 14 (45.2%) were school students, 4 (12.9%) university students, 4 (12.9%) housewives, 1 (3.2%) self-employed, 4 (12.9%) educational staff members, 2 (6.5%) office workers, 1 (3.2%) member of the military sector and 1 (3.2%) restaurant staff (9 people without jobs less than 6yr old.

All subjects had allergic history including asthma 10 (25%), allergic rhinitis 8 (20%), urticaria 19 (47.5%) and other diseases 3 (7.5%). *Dermatophagoides pteronyssinus* was more frequent than *De. farinae* (98% vs. 83%). The condition of patients’ home of 31 cases is recorded in Table 1.

**Table 1.** Frequency distribution of residential home in terms of considering sun light provision in positive prick test and negative prick test groups

**Prick test**	**Prick test positive Number (%)**	**Prick test negative Number (%)**	**P value**
**sunshine is fully provided**	12 (60)	19 (95)	0.02
**sunshine is not provided**	8 (40)	1 (5)	

Fisher exact test showed that there was a significant difference between two groups in terms of sunshine provision which had fewer mites (P= 0.02). The kind of carpets used in the living room is mentioned in Table 2.

**Table 2.** Frequency distribution of carpet kinds in the living rooms of patients suffering from atopic dermatitis in the positive prick test and negative prick test groups

**Prick test bedrooms' carpet**	**Prick test positive Number (%)**	**Prick test negative Number (%)**	**P value**
**Hand-made**	5 (25)	0(0)	0.04
**Machine-made**	15 (75)	20 (100)	

Fisher exact test showed a significant differences between using hand-made carpets and machine-made carpets in patient’s living rooms (P= 0.04).

### Experimental results

The results of direct observation of mites are recorded in Table 3.

**Table 3.** Frequency distribution of direct observation of mites in patients’ bed sheet carpets in positive and negative prick tests

**Prick test direct observation of mites**	**Prick test positive Number (%)**	**Prick test negative Number (%)**	**P value**
**bedding sheets**	7 (35)	0 (0)	0.008
**bedrooms’ carpet**	6 (30)	0 (0)	0.02
**living room’s carpet**	3 (15)	1(5)	0.60

## Discussion

Briefly, the prevalence of allergic diseases due to household arthropods have significantly increased in the recent last decades, because people spend most of their time in their home environment and according to the modern lifestyle, houses are warmer and filled with a lot of furniture and not enough air-conditioning is provided (Yong and Jeong 2009). In this study, we determined the abundance of dermatophagoides mites in the living house of patients suffering from Atopic dermatitis.

House dust mites are the most common allergens in closed environments (Voorhorst et al. 1964). The first determinant factor of dust mite number is humidity and the most important amount of relative humidity in a house for the maximum rate seems to be 60 % in 21 °C and 75% in 15.5 °C (Platts-Mills and Chapman 1987). During sampling, the amount of humidity of all houses was recorded. The mean humidity in houses was 34.4% (33.6±11.3). In lack of humidity every kind of bed sheets, pillows and furniture are safe for patient. Regular cleaning and vacuuming cause to decrease mites. Mature mites will die in 5 to 11 days if they are exposed to the environmental temperature of 25 °C to 35 °C with the constant relative humidity of 40% to 50% because of dehydration (Arlian and Platts-Mills 2001). Dust mites are limited in the area near the Caspian Sea, while in other areas due to the seasonal variations of temperature and humidity; mites are not able to grow well (Fereidouni et al. 2013).

According to our study at houses which sunshine is fully provided, fewer mites were found which showed that changing life style would help exposing allergenic mites.

We concluded long lint carpet could add the number of mites. Handmade carpets had more mites than machine made ones. This is according to the study of Mihrshahi et al. (2002) residential houses in Sidney in Australia where wool carpets with long lint (hand-woven) are the best habitats for mites and they can survive in these carpets for a long period of time (Mihrshahi et al. 2002).

Result of the direct observation of mites in bed sheet was 35% at old woolly bed. This is similar to the result of two studies conducted in Sidney and Copenhagen and confirmed that the most important predictor factor for the high density of allergen mites was old wool beds (used for more than 2yr) (Mihrshahi et al. 2002, Sidenius et al. 2002). In this study, the minimum number of mites (1 to 2 mites in 100g of dust) was obtained from the residential houses of patients with atopic dermatitis. In comparison with other studies in other regions, this number is small.

We found more mites in old residential homes. This is in accordance to a study at houses in Sydney in Australia, which revealed that the ages of buildings provides the most significant theory about the density of mite allergens in beds and on floors of bedrooms, and older houses are full of dust (Mihrshahi et al. 2002), so they can provide a big source for dust mites (Arbes Jr et al. 2003).

## Conclusion

According to the present study, 55% of residential places of prick test positive patients and only 5% of residential home of prick test negative patients were positive for mite. Sunshine provided home had fewer mites than home where sunshine is not provided. Prick test positive patients used hand-made carpets more than machine made ones. In positive prick test patients, mites were found in bed sheet and bedrooms carpet more than negative prick test patients’ sheets and carpets.
